# Open Issues and Practical Suggestions for Telemedicine in Chronic Pain

**DOI:** 10.3390/ijerph182312416

**Published:** 2021-11-25

**Authors:** Marco Cascella, Franco Marinangeli, Alessandro Vittori, Cristina Scala, Massimo Piccinini, Alessandro Braga, Luca Miceli, Renato Vellucci

**Affiliations:** 1 Division of Anesthesia and Pain Medicine, Istituto Nazionale Tumori—IRCCS—Fondazione Pascale, 80131 Napoli, Italy; m.cascella@istitutotumori.na.it; 2 Department of Life, Health and Environmental Sciences, University of L’Aquila, 67100 L’Aquila, Italy; francomarinangeli@gmail.com; 3 Department of Anesthesia and Critical Care, ARCO, Ospedale Pediatrico Bambino Gesù IRCCS, 00165 Rome, Italy; 4 UOC Anesthesia, Intensive Care and Pain Therapy, Senigallia Hospital, 60123 Ancona, Italy; scalacri@virgilio.it; 5 Anesthesia, Critical Care, Palliative Medicine and Pain Therapy Service, L’Aquila ASL1 Abruzzo, 67100 L’Aquila, Italy; massimo.piccinini@yahoo.it; 6 Grunenthal, Italia srl, 20019 Milan, Italy; alessandro.braga@grunenthal.com; 7 Department of Clinical and Experimental Pain Medicine, IRCCS CRO of Aviano, 33081 Aviano, Italy; luca.miceli@cro.it; 8 Pain and Palliative Care Clinic, University Hospital of Careggi, 50121 Florence, Italy; renato.vellucci@gmail.com

**Keywords:** chronic pain, telemedicine, healthcare delivery, health-related quality of life, functionality

## Abstract

Telemedicine represents a major opportunity to facilitate continued assistance for patients with chronic pain and improve their access to care. Preliminary data show that an improvement can be expected of the monitoring, treatment adherence, assessment of treatment effect including the emotional distress associated with pain. Moreover, this approach seems to be convenient and cost-effective, and particularly suitable for personalized treatment. Nevertheless, several open issues must be highlighted such as identification of assessment tools, implementation of monitoring instruments, and ability to evaluate personal needs and expectations. Open questions exist, such as how to evaluate the need for medical intervention and interventional procedures, and how to define when a clinical examination is required for certain conditions. In this context, it is necessary to establish dynamic protocols that provide the right balance between face-to-face visits and telemedicine. Useful tips are provided to start an efficient experience. More data are needed to develop precise operating procedures. In the meantime, the first experiences from such settings can pave the way to initiate effective care pathways in chronic pain.

## 1. Introduction

Telemedicine has been defined as the use of electronic technologies for communication and information of patients, to provide the public with remote healthcare services [1]. Although it has existed for more than two decades, its implementation has been limited for many years, until the emergency during the COVID-19 pandemic. This catastrophic event has promoted attempts to provide chronic patients with adequate care despite restrictions to in-presence activities [2]. The definition of adequate care pathways for chronic pain will need long clinical trials as the condition is complex and many different cases must be faced. As an example, patients with chronic post-surgical pain who have a certain diagnosis, a complex condition, and need careful and prolonged follow-up, and can benefit from the assistance through remote services. Recent experiences have suggested that telemedicine can improve access to care, facilitate continuity of care, allow better resource efficiency, and lower costs, compared with traditional in-person hospital or ambulatory visits [1,3,4]. Improved access to care is expected to enhance timely adjustment of therapy, and improved adherence, which could reduce the progression toward reduced functionality of patients with chronic pain [5]. Appropriate uses of telemedicine for patients with chronic pain have recently been described. It has opened a promising field of activity, although a business case analysis would be needed in each prospective application [2,6].

On these premises, best practice approaches for telemedicine programs in chronic pain need to be suggested, to enable clinicians to provide and patients to benefit from remote assistance.

This article is based on the direct experience of a group of clinicians and attempts to provide a framework to prepare physicians, patients with chronic pain, and caregivers to use telemedicine with satisfactory results.

## 2. The Complexity of the Patient with Chronic Pain

According to the International Association for the Study of Pain (IASP), chronic pain is commonly defined as persistent or recurrent pain lasting more than 3 months or beyond normal tissue healing [7]. It has been recognized as a real disease associated with multiple adaptations in the nervous, endocrine, and immune systems [8]. Consequently, chronic pain is a complex multidimensional experience severely compromising the patient’s health-related quality of life (HRQoL), often limiting the ability to work, sleep, and affecting social interactions with friends and family [2,9]. Reduced functionality, emotional imbalances, and social isolation are frequently associated complaints and may exacerbate each other in a vicious circle that compromises the HRQoL and induces a progression toward disability [10,11]. Furthermore, chronic pain is often associated with multimorbidity. In particular, many patients with chronic pain have other comorbidities, such as depression, cardiovascular and pulmonary diseases, diabetes mellitus, and cancer [12,13]. Notably, proper pain control may be extremely challenging in multi-morbid patients as comorbidities and their treatments can increase the risk of side effects of analgesics and thus limit the applicability of disease-specific clinical guidelines [14]. On the other hand, chronic pain is an independent risk factor for mortality in people with other co-morbidities [15]. Moreover, patients with chronic pain require multidisciplinary, continuous, and skilled management, which may challenge healthcare system organizations. Difficulties with the traditional models of care, with in-person patient visits to their physicians inherently leading to delayed care, have further cumulated during the COVID-19 pandemic and the need to structure new care pathways to ensure appropriate treatment of chronic pain patients, became even more prominent.

The first and necessary steps for the management of chronic pain are diagnosis and assessment of all pain dimensions. Given the 11th International Classification of Diseases (ICD-11) introduction, chronic pain will be classified into primary and secondary pain. Chronic primary pain can be conceived as a disease. This is a definition, which applies to chronic pain syndromes that are best conceived as health conditions in their own right [7]. Chronic secondary pain syndromes are linked to other diseases as the underlying cause, for which pain may initially be regarded as a symptom. Secondary pain includes cancer-related, post-traumatic and postsurgical, neuropathic, visceral, musculoskeletal, and headache/orofacial pain [7]. In many cases, the secondary chronic pain may continue beyond the successful treatment of the initial cause; in such cases, the pain diagnosis will remain, even after the diagnosis of the underlying disease is no longer relevant. This distinction is particularly important because it conditions the clinical, diagnostic, and therapeutic approaches. For example, in the cancer patient, close monitoring with close controls is mandatory when opioids are used. Moreover, in primary chronic pain (e.g., fibromyalgia) a combined approach with the collaboration of different professional figures may be necessary.

## 3. Approaches of Telemedicine for Patients with Chronic Pain

Telemedicine has been defined as the use of electronic technologies for communication and information to provide the public with healthcare services at a distance. More recently, the American Medical Association defined “telehealth” as a general group of modalities allowing: (1) real-time audio and visual connections between patients and physicians in different locations; (2) image and data collection storage and sharing for later interpretation; (3) remote patient monitoring tools, including mobile health (mHealth) tools, wearables, and devices; and (4) virtual check-ins through voice-only patient portals, messaging technologies [16]. The Italian Ministry of Health produced guidelines for telemedicine and defined the televisit as “a health act in which the doctor interacts remotely with the patient”. The definition specifies that “the health act of diagnosis that arises from the visit may give rise to the prescription of drugs or treatments” [17].

Thus, the terms “telemedicine” and “telehealth” are commonly used interchangeably and encompassed in the set of tools called “telecare” [18]. Telecare includes several possible modalities and activities, such as archiving and sharing medical images or biosignals (e.g., in the fields of radiology, or dermatology), telemonitoring, and real-time interactive services. The latter modalities include a variety of services, such as telenursing, telepharmacy, telerehabilitation, emergency counseling, and mostly online consultation via remote visits and/or multi-professional teleconsulting.

## 4. Online Consultation Pathway

**Figure 1:**
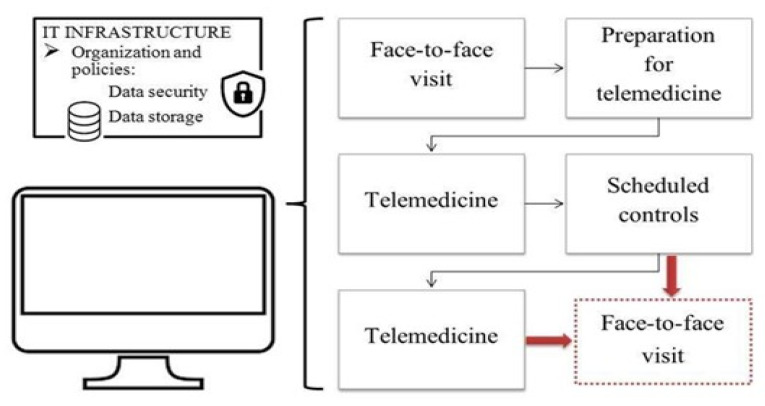
Telemedicine pathway for addressing chronic pain.

A remote system is a great opportunity to improve access to care and continuing assistance, which may help to personalize treatments and to increase adherence. To fulfil these objectives, it is necessary to structure a defined pathway. It is divided into a series of technical processes (information technology infrastructure) and operational phases (preparation, execution, scheduling of controls) (Figure 1).

The information technology infrastructure must allow all organizational phases (reservations, contacts, links for connection, and data collection) and guarantee data security. Operation includes a first visit usually performed in person. This face-to-face assessment is followed by the preparation for telemedicine (legal and regulatory issues, patient information, technical issues). Later, telemedicine is performed, and scheduled controls are programmed. New in-person visits can be required (e.g., to carry out minimally invasive procedures). IT, information technology.

### 4.1. IT Infrastructure Functioning

The COVID-19 pandemic and the need to provide alternative ways for the in-person visit has led to the creation of a wide range of information technology (IT) infrastructures. On the market, there are systems which have different complexities (and costs). In general terms, the platforms consist of an operating system for the management of the whole service, devices (e.g., laptops), and an integrated software system (software modules) for sending documents, reports, and imaging. In brief, the IT infrastructure must allow all organizational phases (reservations, contacts, links for connection, and data collection) and guarantee data security and privacy.

### 4.2. Operational Phase: First Face-to-Face Visit

Since programs of telemedicine should be based on good interpersonal relationships, a first visit should usually be performed in-person to:

- Establish a relationship with the patient, which is necessary for long-term reliance.
- Obtain a diagnosis of chronic pain.
- Assess and measure pain and HRQoL.
- Enable the physician to perform assessment maneuvers.
- Prepare the telemedicine visit.

An in-person contact provides the necessary and reciprocal confidence for further remote relationships. Indeed, patients must be evaluated for their ability to use telemedicine before a program is established. Most of the information infrastructures available for telemedicine require that patients and/or caregivers simply need to have an email address and a smartphone, iPad/tablet, or a personal computer with a camera and speakers. Based on the in-person visit, a remote follow-up and monitoring schedule can be prepared.

### 4.3. Operational Phase: Remote Follow-Up

For remote follow-up it is important to collect the right parameters that can be obtained remotely. All the different aspects of chronic pain must be monitored; pain intensity, therapeutic adherence, sleep quality, movement functionality, emotionality, and working abilities. Patients and caregivers need training in order enable them to focus on the relevant topics during the telemedicine visit; the physician will guide the visit and choose the relevant area to be investigated in the situation.

#### 4.3.1. Clinical Assessment

The remote evaluation involves the study of the patient’s medical record (imaging, laboratory tests, other documents) and the clinical–diagnostic phase. A comprehensive pain assessment is a crucial step in the management of a patient with pain. As already stated, chronic pain is a multidimensional experience resulting in impaired functioning in daily life and reduced quality of life and well-being of the patient, as it can be observed for chronic low back pain (cLBP) [19]. We suggest that physicians could assess pain severity mainly using the parameters of pain intensity, pain-related distress, and functioning.

Generally, assessment tools for telemedicine should be validated, and suitable; moreover, the same tools should always be used. In addition, other instruments can be used, for the objective evaluation of distress and functionality through web-based use. Based on the authors’ experience, a combination of unidimensional and multidimensional tools can be adopted. The numerical rating scale (NRS) may easily be used by patients to assess pain intensity. The Brief Pain Inventory (BPI) is a validated, simple, and self-completed questionnaire (visual administration) that evaluates not only pain intensity, but also functionality, and provides long-term monitoring in patients with progressive conditions. The tool is reliable and valid for many clinical situations (e.g., cancer pain, and non-cancer pain conditions) and across cultures and languages. Functionality can be assessed by an ecological matrix scale [20,21,22], which considers the outer environment and the personality structure of the patient, the motives, the personal expectations, and needs, and helps the patient and the physician to identify treatment objectives that may be satisfactory for the patient.

In the setting of chronic low back pain, the Oswestry Disability Index and the Roland-Morris disability questionnaire may be useful for the functional evaluation of low back pain [22,23].

Other indices are available, such as the Low Back Pain Rating Scale (LBPRS), as an example, the Progressive Isoinertial Lifting Evaluation (PILE), and the Quebec Back Pain Disability Scale (QBPDS) [24]. In this respect, clinicians should also evaluate the capability of their patients to perform daily activities, the patient’s emotional status, and their strength. Up to now, different proper instruments are available to evaluate HR-QoL, such as the EQ-5D from the EuroQol Research Foundation, and the short form (SF-12 scale) of the 36-item Health Survey instrument are available [25,26].

In addition to the evaluation through tools, all the anamnestic elements must be collected. Even if at a distance, the clinician will have to investigate the clinical elements of the painful symptomatology: location, intensity, triggering factors, therapies carried out, and comorbidities. Peculiar aspects, such as breakthrough cancer pain, drug effects, and clinical conditions, which may affect the use of particular categories of drugs (e.g., organ damage), must be evaluated.

Finally, the recommendations issued by the Initiative on Methods, Measurement, and Pain Assessment in Clinical Trials (IMMPACT) can also be used. A common tool would be used to evaluate the validity of telemedicine models. Notably, six core outcome domains were recommended by IMMPACT including pain, physical functioning, emotional functioning, participant ratings of global improvement, symptoms and adverse events, and patient disposition (adherence to the treatment regimen, reasons for withdrawal from treatment) [27]. A systematic review showed that eHealth and mHealth interventions had significant effects on multiple short- and intermediate-term outcome measures recommended in the IMMPACT guidelines [28].

#### 4.3.2. Outcomes, Therapy, and Re-Evaluation

The televisit can give rise to different outcomes; it is possible to achieve clinical stability within the already known diagnostic framework. It can bring out the need for urgent access to diagnostic or therapeutic services, which require the patient to have a face-to-face consultation with the pain specialist. The third scenario includes the need for further examination to have a diagnosis, which the specialist will manage with the prescription of the necessary services.

During the televisit, the previous therapy can be either confirmed or changed; in this case, the specialist prescribes the drugs and sends the prescription to the patient as agreed with him and his caregiver. Although the primary objective of treatment should be control of pain intensity, functional recovery and general wellbeing are the overall aims of the patient’s management. This means that pharmacological and non-pharmacological treatments must be considered, and that tailored objectives are to be pursued according to the expectations and needs of each patient [20,21,22].

If it is impossible to reach a diagnostic or therapeutic conclusion, the doctor will propose the execution of a further check-up in the times and the ways appropriate to the clinical situation, and the follow-up pathway will be planned. In some cases, possible adverse events may be mentioned by the physician, asking the patients whether any of them occurred in the last period; this may prove a simple and effective method to detect tolerability issues. Under certain circumstances, such as drug side effects, need for a physical examination or interventional procedures, an in-person visit may be necessary.

The remote visits should be tightly planned to ensure that monitoring can be performed adequately, and therapy can be adjusted. The data collected and outcome of the televisit will need to be recorded as a routine medical visit and filed according to local customs.

## 5. Open Issues and Suggestions

When developing a telemedicine plan for the care of patients with chronic pain, a number of challenges need to be considered. These include the risk of adverse effects of drugs prescribed in a remote visit, the correct management of patients with advanced age, cognitive impairment, emotional frailty, and the need to alternate remote and in-person visits. To address these challenges, some suggestions may be useful. Table 1 presents the recommended steps for the implementation of a telemedicine service for patients with chronic pain.

Prerequisites for remote care systems include correct and exhaustive information about legal issues and regulation, availability of suitable technical equipment, and specific medical skills for the remote management of patients with chronic pain so that a correct assessment is performed despite the lack of a physical inspection. Specifically, all local requirements for healthcare must be fulfilled by remote systems, as well as by traditional in-presence organizations, and some technical solutions must be found to this aim. Therefore, first of all, knowledge of regulation is needed so that medico-legal problems are detected and addressed, and then rules must be adapted to remote systems [29]. The main legal issues to be faced are related to data protection, privacy, and delivery of reports and prescriptions. Several online platforms are available, which respect such requirements.

A basic suitable technical equipment for the physician and the patient includes an efficient connection to the internet, a digital device (usually a PC) with a webcam, and a customized web-based platform. In addition, to set up a telemedicine system, a large proportion of the target population needs to have sufficient skills to use the proposed web-based platform.

This means that very simple digital tools are to be preferred. Common social media are often used, to facilitate patients and caregivers, but they would not fulfill privacy and data protection requirements. The current reference guidelines for telemedicine are obsolete [30]; some of those, for example, date back to the 1980s and were not followed by later recommendations [31,32,33,34]. Privacy regulations, technological opportunities, and problems related to the pandemic are open issues that must be urgently addressed. The use of validated and protected platforms available through public or private healthcare providers could help respond to these issues.

As an example, the Regional Health Service in Tuscany, Italy, made a digital platform for televisits available to all specialists, in June 2020. The online visit requires a PC (with Windows 7 or IOS 11, or later versions), or smartphone or tablet (with Android 5.0 or later version), or iPhone or iPad (with IOS 11 or later version). The platform was mainly used by diabetologists, rheumatologists, and cardiologists, and registered up to 5000 visits in the first month of activity. This platform is extremely user friendly, is linked to the online clinical records, and with the regional health booking service; digital prescriptions are delivered by a preexisting system, while a final report of each visit is provided on the platform, according to specific rules set by the regional healthcare system.

The last suggestion on this point is that patients and/or caregivers must be exhaustively trained to be able to use the telemedicine system.

In summary, before the initiation of the telemedicine program, the medical activities must be carefully planned and scheduled. Online performances will be very fast, and waiting times are very limited online; so, everything must be ready beforehand. In addition, it is important to identify the parameters deemed suitable for remote visits on one end and for telemonitoring on the other one. The patient and/or the caregiver must be selected and, if accepted, prepared. Selection will be based both on medical (as an example, chronic, stable conditions are more suitable than lately diagnosed, progressing patients) and cognitive qualities, and personal features. The patient/caregiver must accept the program and feel it as an opportunity for improved management, continuing assistance, and access to care. If a caregiver is necessary, the same person should be present at all remote visits, and the same person should oversee assessments for monitoring. If the patient is a child, special precautions should be used for an effective protection of her/his rights. A relationship between the patient and/or caregiver and the pain specialist should already exist, with a good therapeutic alliance. A first in-person visit will usually be performed to obtain a diagnosis, to prepare the patient, and state a reciprocal reliance. This phase is followed by telemonitoring and scheduled remote control visits. A strict and punctually respected schedule of the program will facilitate its long-term continuation.

Special attention is necessary for the organization of the clinical assessment. Firstly, suitable tools must be identified. As previously mentioned, simple tools are to be offered; NRS, BPI, self-evaluation numerical scales, and ecologic matrix scales may be used. The assessment frequency must be stated in advance and explained to the patient. A telemedicine system for chronic pain will improve continued assistance but does not usually provide an emergency service. Patients must be informed of the aim of the system and should know what to do in case of adverse events or serious pain episodes. If around-the-clock assistance is necessary according to the patient’s conditions, a phone triage should be available to deliver primary information and refer the patient to the correct health operator. Finally, a telemonitoring program will be efficient if the patient and/or caregiver are empowered; so, great care is due to instruction, and information about the disease and the treatment.

During the online evaluation, the physician will not be able to perform physical maneuvers which are commonly used for in-presence assessment, but some simple ones may be proposed to the caregiver. Each visit will be mainly based on the evaluation of the report by the patient and the revision of data collected during the telemonitoring. The physician should develop his/her listening ability as much as possible, and empathy. Punctuality, respect of visit duration, and the ability to listen to the patient and/or caregiver are very important. After each visit a medical report must be delivered; predisposed forms can be designed, and an identical delivery system, such as email, should be routinely used. As an example, patients with musculoskeletal chronic pain are very likely to benefit from an assiduous follow-up performed with a multidimensional assessment made possible and affordable by the introduction of telemedicine [35,36,37].

## 6. Limitations

We acknowledge that several problems limit the possibility of achieving our aim of providing useful hints for the implementation of telemedicine for chronic pain. No evidence is available on the efficacy of this model. Regulation is widely different worldwide, and this makes it impossible to address local issues. As an example, medical responsibility is criminal and not only civil in Italy. In addition, certified online platforms are very different from each other. All these discrepancies make it difficult to design the many care pathways that are needed to address a complex condition such as chronic pain.

## 7. Conclusions

Telemedicine seems to be promising for the efficient management of patients with chronic pain. This approach can deliver tailored pain management, providing improved access to health services and creating and maintaining a therapeutic alliance in the long term. Nevertheless, the effects of therapies provided via telemedicine on pain and pain-related conditions, such as disability, depression, and anxiety, are promising but not well documented yet. Furthermore, when approaching remote assistance for chronic pain, several issues are to be faced such as accurate diagnosis, assessment, monitoring and need to change treatment. Consequently, the implementation of new web-based systems for the management of chronic pain needs further evaluation and well-structured pathways must be necessarily built. Finally, as privacy regulation is incomplete worldwide, clinicians are bound to be extremely cautious about respecting the patient’s rights.

## Figures and Tables

**Table 1:** Some recommended actions for the management of a telemedicine system.

Steps	Recommended Actions	What Is Needed
Preparation of a telemedicine system	Legal and regulatory issues	Acquire information about: Local rules for requirements Data protection Remote informed consent Clinical report and prescription release
	Technical equipment	An efficient connection to the internet A digital device with a webcam Web-based platform
	Medical skills	Identify the phases of the visit Identify the parameters suitable for remote assessments of pain intensity, personal requirements, functionality, sleep quality, adverse events, treatment adherence Identify diagnostic maneuvers that can be performed by caregivers in your place
Initiation of a telemedicine program	Prepare the patient	An in-person visit is usually necessary before any telemedicine program, to: Diagnose the condition Assess pain Explain the program Establish a relationship
	Schedule the visits beforehand	Both the clinician and the patient need to know how much time is dedicated to the visits, to have the opportunity to continue the program long-term
Monitoring	Provide the patient with tools and instructions Schedule the assessment frequency Provide instructions for emergencies	Dedicated digital platforms with assessment scales
Visits	Have a schedule and confirm each date Be aware that it may be necessary to alternate remote and in-person visits	Be punctual Let the patient speak first Control times Release a report

Note: BPI, brief pain inventory.
